# Targeting PINK1 Using Natural Products for the Treatment of Human Diseases

**DOI:** 10.1155/2021/4045819

**Published:** 2021-10-30

**Authors:** Yan-Qin Li, Fan Zhang, Li-Ping Yu, Jian-Kang Mu, Ya-Qin Yang, Jie Yu, Xing-Xin Yang

**Affiliations:** ^1^College of Pharmaceutical Science, Yunnan University of Chinese Medicine, 1076 Yuhua Road, Kunming 650500, China; ^2^Yunnan Key Laboratory of Southern Medicine Utilization, 1076 Yuhua Road, Kunming 650500, China

## Abstract

*PINK1*, also known as *PARK6*, is a *PTEN*-induced putative kinase 1 that is encoded by nuclear genes. PINK1 is ubiquitously expressed and regulates mitochondrial function and mitophagy in a range of cell types. The dysregulation of PINK1 is associated with the pathogenesis and development of mitochondrial-associated disorders. Many natural products could regulate PINK1 to relieve PINK1-associated diseases. Here, we review the structure and function of PINK1, its relationship to human diseases, and the regulation of natural products to PINK1. We further highlight that the discovery of natural PINK1 regulators represents an attractive strategy for the treatment of PINK1-related diseases, including liver and heart diseases, cancer, and Parkinson's disease. Moreover, investigating PINK1 regulation of natural products can enhance the in-depth comprehension of the mechanism of action of natural products.

## 1. Introduction

*PINK1* (*PARK6*) is a PTEN-induced putative kinase 1 and one of the most abundant human protein kinases [1]. PINK1 was first discovered nearly 20 years ago due its dysregulation in ovarian cancer and soon after was reported to be important in the development of Parkinson's disease [2]. To date, PINK1 has been implicated in a range of pathologies including liver, heart, and skeletal muscle injury due to its role in mitochondrial quality control. More recent studies indicate that natural products can relieve human diseases through their ability to regulate PINK1 activity.

This paper reviews the structure and function of PINK1, investigates the relationship between PINK1 and human diseases, and highlights the regulatory effects of natural products on PINK1. We herein propose that the identification of PINK1 regulators from natural products represents an attractive strategy for the treatment of PINK1-related diseases. Furthermore, future in-depth and comprehensive analysis of the underlying mechanism(s) of action of natural products and the role of PINK1 in these processes may further reveal its role during disease development.

## 2. PINK1 Structure

**Figure 1:**
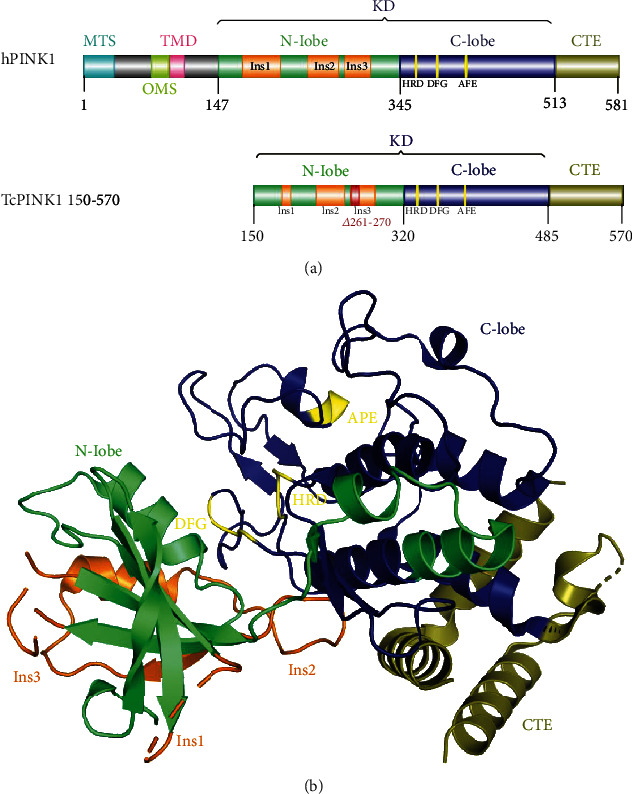
Structure of PINK1. (a) Domain architecture of human PINK1 (hPINK1) showing MTS (1-34), OMS (74-93), TMD (94-110), KD (147-513), and CTE (514-581) domains. Location of the N-lobe (147-345), C-lobe (346-513), three insertions: Ins1 (174-215); Ins2 (244-277); Ins3 (284-312), the catalytic motif HRD (360-362), and activation loop motifs: DFG (384-386) and APE (415-417). MTS: mitochondrial targeting sequence; OMS: outer membrane localization signal; TMD: transmembrane domain; KD: kinase domain; CTE: C-terminal extension; Ins: insertion. Created with IBS [60]. Domain architecture of the crystal structure of Tribolium castaneum PINK1 (TcPINK1, PDB ID: 5OAT) [3] is shown in the lower panel. KD (150-486), CTE (487-570), N-lobe (151-320), C-lobe (321-486), Ins1 (182-192), Ins2 (221-253), Ins3 (260-288), HRD (335-337) DFG (359-361), and APE (390-392). *Δ*261-270 indicates deleted residues during the crystallization of TcPINK1. (b) Crystal structure of TcPINK1 (PDB ID: 5OAT) [3]. N-lobe, C-lobe, CTE, Ins1, Ins2, Ins3, HRD, DFG, and APE are colored as in (a); disordered regions are shown in dashed lines. Generated with PyMol [61].

Human *PINK1* (hPINK) is located on 1p35-p36 of chromosome 1 and has 8 exons with a length of ~18 kB. hPINK1 is 581 amino acids in length and can be divided into several domains (Figure 1(a)). Its N-terminus possesses a mitochondrial targeting sequence (MTS), an outer membrane localization sequence (OMS), a transmembrane domain (TMD), and a nonconservative region. The middle region is composed of 367 amino acids that are highly homologous to serine/threonine kinases of the calmodulin family, forming a kinase domain (KD) with an N-lobe containing three insertions (Ins1, Ins2, and Ins3), a C-lobe containing a catalytic motif (HRD), and two activation loop motifs (DFG and APE). The C-terminal extension (CTE) forms a regulatory domain composed of 69 amino acids [2–6].

The 3D structure of hPINK1 has remained elusive due to its low levels of expression, susceptibility to degradation, and poor solubility. This restricts protein production and manipulation for structural studies by X-ray crystallography and nuclear magnetic resonance (NMR). However, the kinase domain of *Tribolium castaneum* PINK1 (*Tc*PINK1) in the apo (PDB ID: 5OAT) [3] and ATP analogue AMP-PNP-bound forms (PDB ID: 5YJ9) [4], in addition to the kinase domain of *Pediculus humanus corporis* PINK1 (*Ph*PINK1) in the Ub-bound form (PDB ID: 6EQI) [5], were successfully crystalized using different strategies. These structures revealed that the PINK1 kinase domain adopts a well-known bilobal kinase fold, and the CTE forms a PINK1-specific extension (Figure 1(b)). The N-lobe of kinase domain contains a five-stranded antiparallel *β*-sheet, an *α*-helix, and three predominantly disordered insertions that distinguish PINK1 from other kinase structures.

The Ins1 varies across species in terms of length and residue identity, whilst Ins3 and the C-distal region of Ins2 are highly conserved. Ins2 interacts with the C-lobe and CTE and may play a regulatory role during PINK1 activation. As the most conserved insertion, Ins3 is directly involved in substrate recognition and binding. The C-lobe of the kinase domain possesses HRD, DFG, and APE motifs with highly conserved conformations and functions. The C-terminal extension is well conserved and regulates PINK1 activity through binding to the regulatory subunit, participating in the recruitment of PINK1-related substrates. PINK1 is not only restricted to the mitochondria, but is also expressed in the cytoplasm [7]. PINK1 participates in mitochondrial quality control, stress responses, and metabolic function.

Under normal physiological conditions, PINK1 is synthesized in the cytoplasm and is imported into the inner mitochondrial membrane, where it is subsequently degraded by proteolytic enzymes. When the membrane potential of the mitochondria dissipates due to mitochondrial damage, the transfer of PINK1 to the inner membrane is blocked, and PINK1 accumulates on the outer mitochondrial membrane. At the same time, Parkin is recruited from the cytoplasm by PINK1 and phosphorylated. Activated Parkin catalyzes the ubiquitination (UB) of mitochondrial proteins, enabling their recognition by connexin. UB proteins associate with yeast autophagy protein 8 family homologous proteins including microtubule-associated protein light chain 3 (LC3) and Golgi body-associated ATPase enhancer on phagocytic membranes to induce mitochondrial autophagy. Finally, the mitochondria-derived autophagosomes fuse with lysosomes to form mature mitochondrial autophagosomes, which subsequently imitate the degradation process.

## 3. PINK1 and Human Diseases

PINK1 is expressed in the brain, heart, liver, and skeletal muscle tissue. Its expression closely correlates with mitophagy regulation during human disease, highlighting its physiological significance.

### 3.1. PINK1 and Parkinson's Disease (PD)

PD is a complex disorder caused by multiple genes and environmental factors [8]. The main pathological phenotypes of PD are due to the age-dependent loss of dopaminergic neurons in the substantia nigra [9]. PD leads to the loss of dopamine neurons by oxidative stress, mitochondrial dysfunction, inflammatory responses, apoptosis, and other pathological mechanisms. Genetic factors play a key role, with *PINK1* mutations leading to autosomal recessive PD. Mitochondrial dysfunction is implicated in sporadic and familial PD [10]. PINK1 maintains mitochondrial function and reduces mitochondrial oxidative stress. When PINK1 expression is suppressed, oxidative stress is impaired, resulting in decreased activity of mitochondrial complex I and excessive generation of oxygen free radicals, which are induced in PD [11]. In addition, PINK1 promotes the removal of *α*-synuclein aggregates and protects neurons from *α*-synuclein-mediated damage [12]. Overexpression of PINK1 also enhances the phosphorylation of Akt at Ser473, with activated Akt protecting SH-SY5Y neuronal cells from various cytotoxic substances including reactive oxygen species (ROS) [13]. PINK1 deficiency leads to the defective differentiation of neural stem cells into astrocytes, which induces neuronal death and/or the abnormal repair of damaged brain tissue, resulting in an increased risk of PD [14]. Small interfering RNAs have also been shown to significantly inhibit the expression of *PINK1* in human dopaminergic cells such as SH-SY5Y, decreasing their viability [15]. Restoring the expression of PINK1 represents a promising strategy for future PD therapies.

### 3.2. PINK1 and Cancer

*PINK1* displays tumor suppressor activity [16]. PINK1 expression is not limited to the brain, but shows an ubiquitous distribution with a subcellular localization that is cell type dependent [17]. Early studies showed that PINK1 is highly expressed in breast, colorectal, and endometrial cancer tissues. In addition, the downregulation of PINK1 inhibits the proliferation, colony forming ability, and migration of cancer cells [16, 18] and increases the sensitivity of tumor cells to stressors [15, 19]. PINK1 promotes tumorigenesis by activating factor-1 receptor-photodynamic kinase 3/Akt signaling [13]. Through parallel high-throughput RNA interference screening, it was found that in the context of DNA mismatch repair defects in cancer, PINK1 silencing led to increased ROS production and subsequent oxidative DNA damage in the nucleus and mitochondria. In cancer patients with DNA mismatch repair defects, the development of drugs that trigger mitochondrial and nuclear oxidative DNA damage, therefore, represents a potential therapeutic approach [20]. The overexpression of PINK1 activates NF-*κ*B signaling and promotes the development of non-small-cell lung cancer (NSCLC). The downregulation of PINK1 can enhance cisplatin- (CDDP-) induced apoptosis in NSCLC cells [18] and makes cervical cancer cells (including HeLa and BT474 cells) sensitive to paclitaxel, significantly increasing cell death [21]. It is however postulated that PINK1 possesses both pro- and anti-apoptotic activity that is dependent on the cell environment [16]. Mitophagy plays a dual role in hepatocellular carcinoma (HCC) development depending on the stage of tumorigenesis [22–24]. Therefore, PINK1 represents a potential target for anticancer therapeutics.

### 3.3. PINK1 and Liver Disease

#### 3.3.1. PINK1 and Metabolic-Associated Fatty Liver Disease (MAFLD)

MAFLD is the most common chronic liver disorder worldwide. MAFLD encompasses a broad spectrum of hepatic disorders [25] that may advance to hepatitis, liver cirrhosis, and liver cancer [26, 27]. The removal of damaged mitochondria through mitophagy is widely regarded as a protective mechanism during long-term MAFLD development. In mouse models induced by a high-fat diet, mitophagy defects are related to a series of phenotypes related to MAFLD [28–30]. The PINK1-Parkin pathway is a major regulator of hepatocyte mitophagy and is key to the cyanidin-3-O-glucoside-mediated alleviation of MAFLD [31]. In mouse models of MAFLD, hepatic ALCAT1 (an acyl-CoA-dependent lysocardiolipin acyltransferase) is significantly overexpressed, whilst its deficiency increases the expression of PINK1 and prevents the onset of MAFLD [28]. Hepatic inflammation can be regulated by autophagy, which limits ROS production and the release of DAMPs (damage-associated molecular patterns) [32]. Inflammation is an important cause of hepatitis [33]. In mouse models of hepatitis, the expression of PINK1 and Parkin are suppressed, leading to impaired mitophagy and increased activation of the NLRP3 inflammasome [33]. Mitophagy inhibition with 3-methyladenine/PINK1-directed siRNA weakens the liraglutide-mediated suppression of inflammatory injury [34]. As such, therapeutic efforts to increase PINK1-mediated mitophagy may not only reverse the hepatic manifestations of MAFLD such as hepatocellular steatosis and injury, but suppress metabolic abnormalities associated with disease progression.

#### 3.3.2. PINK1 and Liver Ischemia/Reperfusion (IR) Injury

I/R injury occurs when cellular damage in an organ is initiated during hypoxia or anoxia and becomes exacerbated upon the restoration of oxygen and tissue pH [35]. The liver is the second largest organ in the body. As inferred from its unique dual blood supply, it is vulnerable to ischemic attack due to its highly aerobic nature [36]. There is evidence that mitophagy functions preserve hepatic function and cell survival post-I/R [37, 38]. In mouse liver IR injury models, mitochondrial biogenesis and PINK1-Parkin-mediated mitophagy are attenuated, whilst pharmacological stimulation that increases the expression of PINK1-Parkin enhances mitophagy and improves the outcome of IR, supporting their protective role in IR-induced liver injury [39, 40]. In addition, when AMPK*α* is activated, PINK1-dependent mitophagy is upregulated, which plays a protective role in I/R injury and liver protection [41]. Future studies are required to advance our understanding of the relationship between mitophagy dysfunction and hepatic I/R injury.

### 3.4. PINK1 and Heart Disease

Heart disease is a public health concern and a primary cause of mortality worldwide. The heart has the highest mitochondrial content of all tissues, in which changes in mitochondrial homeostasis severely impact its function [42]. Mitochondrial function and quality control are essential for myocardial contraction [43]. PINK1 can prevent abnormal ROS production and can prevent cardiomyocyte apoptosis. PINK1 knockout mice show spontaneous age-dependent cardiac hypertrophy. PINK1 deficiency alters the mitochondria transmembrane potential (*Δψ* m) in cardiomyocytes, and the expression of PINK1 in the left ventricle of patients with end-stage heart failure is significantly reduced [42]. PINK1 deficiency disrupts mitophagy mediated by PINK1, leading to the inactivation of mitophagy, a loss of myocardial tissue movement, and the development of cardiac hypertrophy [44]. Recent studies reveal new protective pathways of myocardial I/R injury. PINK1 overexpression alleviates hypoxia-reoxygenation-induced cell damage in H9c2 cells through the phosphorylation of TNF receptor-associated protein 1 (TRAP-1) [45]. E2F1 is essential for heart function and affects the metabolism of cardiomyocytes. The E2F1/miR-421/PINK1 axis regulates mitochondrial fracture and cardiomyocyte apoptosis. E2F1 knockout reduces miR-421 levels and promotes PINK1 expression to inhibit mitochondrial damage, apoptosis, and myocardial infarction [46]. AMPK*α*2 in cardiomyocytes phosphorylates PINK1 at ser495 and activates PINK1-Parkin-mediated mitophagy to prevent heart failure [47–49]. It is also reported that specific signaling molecules protect the heart from I/R injury by enhancing PINK1-dependent mitophagy. These signaling molecules include thyroid hormone postconditioning (THPostC), tumor susceptibility gene 101 (TSG101), phosphatase and tensin homolog (PTEN), and apurinic/apyrimidinic endonuclease 1 (APE1) [50–53]. The regulation of PINK1 expression and enhanced PINK1-dependent mitophagy should form the focus of future treatment strategies for heart disease.

### 3.5. PINK1 and Skeletal Muscle Injury

Skeletal muscle is the most abundant tissue in the human body, accounting for 40% of the total body weight. Due to the role of skeletal muscle in exercise, respiration, and energy metabolism, its dysfunction or degradation seriously impacts human health. Mitophagy maintains the homeostasis of skeletal muscle cells [54], but excessive mitophagy can damage skeletal muscle mitochondria [55]. The PINK1-Parkin axis is related to exercise-induced mitochondrial degeneration [56], and in patients with sporadic amyotrophic lateral sclerosis (ALS), the expression of PINK1 is significantly reduced [57]. Traumatic freezing injury leads to mitochondrial dysfunction in skeletal muscle. During this condition, PINK1 expression doubles and mitophagy enhances the clearance of inactivated mitochondria posttrauma [58]. Taken together, the expression of PINK1 can activate mitophagy to clear damaged mitochondria and protect skeletal muscle cells. However, during heavy load exercise, the structure and quantity of skeletal muscle mitochondria are damaged, and a large number of autophagosomes form. During this time, PINK1 and Parkin levels in the mitochondria are significantly elevated which excessively activates mitophagy, leading to mitochondrial damage in skeletal muscle. In models of exhaustive exercise-induced fatigue in mice, *Rhodiola crenulata* oral liquid (RCOL) reduced the expression of PINK1 and Parkin, highlighting that its antifatigue effects are mediated through the inhibition of mitophagy [59]. These results indicate that moderate exercise training can increase PINK1 expression and activate autophagy, which is conducive to maintaining skeletal muscle homeostasis. When the body is exposed to adverse stress, abnormal increases in PINK1 lead to excessive autophagy, abnormal mitochondrial function, and decreased cell viability.

## 4. Regulation of PINK1 by Natural Products

Natural products remain a highly significant source for drug/nutrients development, because of the novel structures, therapeutic abilities, and certain unique pharmacological effects of the chemical substances in natural products. Natural products are widely used to prevent, treat, and diagnose diseases and perform rehabilitation and healthcare. They mainly originate from natural plants, animals, minerals, and their processed products. Both of the monomers and mixtures can be used for the treatment of diseases. With the societal development, the need for disease prevention, medical treatment, rehabilitation, and health maintenance is increasing. Thus, the application of natural products has received unprecedented attention.

Given its roles in multiple tissue types, PINK1 has emerged as a key drug target, with natural products that regulate its expression holding promise for therapeutic interventions. Some natural products (including mixtures and monomers) that regulate PINK1 effectively alleviate its associated diseases (Tables 1–5), including but not limited to liver disease, cancer, and myocardial injury. More in-depth assessments of the constituents of natural products that regulate PINK1 function may further reveal their underlying utility for PINK1-associated disease.

## 5. Conclusions

**Figure 2:**
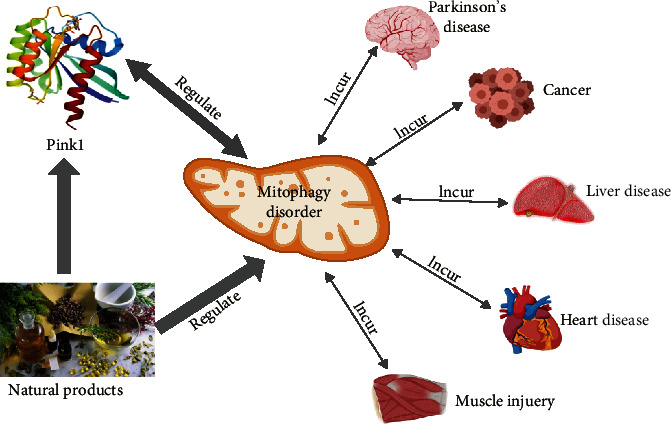
Regulation of PINK1 by natural products for the treatment of human disease.

PINK1 participates in mitochondrial quality control, stress responses, and metabolic signaling. It is also a key regulator of mitophagy. PINK1 dysfunction leads to mitochondrial dysfunction and subsequent disease development, including PD, cancer, liver disease, heart disease, and skeletal muscle injury. It has been reported that some natural products display their therapeutic effects through PINK1 regulation (Figure 2). However, only a few have become clinical drugs for treating patients. It is necessary to explore the regulatory effects of natural products on the expression/activity of PINK1 and its related mechanisms to identify novel compounds that can restore PINK1 function in disease states. In addition, the monomers that regulate PINK1 in many natural extracts remain undefined, and further studies are warranted to assess their utility for the treatment of human disease. With the deepening of research, it is believed that more natural products that can regulate PINK1 may be used to treat diseases, which is of utmost importance.

## Figures and Tables

**Table 1:** Neuroprotection by natural products through PINK1 regulation.

Type	Natural products	Disease	Experimental models
Mixture	Da-Bu-Yin-Wan and Qian-Zheng-San [62]	Parkinson's disease	PINK1 knockdown and 1-methyl-4-phenyl-1,2,3,6-tetrahydropyridine induction in SH-SY5Y cells
	*Eucommia ulmoides* Oliver leaf extracts [63]	Parkinson's disease	1-methyl-4-phenyl-1,2,3,6-tetrahydropyridine-induced zebrafish model
	Grape skin extracts [64]	Parkinson's disease	Drosophila melanogaster model of Parkinson's disease combined with *PINK1* loss-of-function mutations
	*Panax notoginseng* saponins [65]	Cerebral ischemia reperfusion injury	Cerebral ischemia and reperfusion rat model
	*Acanthopanax senticosus* extracts [66]	Parkinson's disease	1-methyl-4-phenyl-1,2,3,6-tetrahydropyridine-induced mouse model
Monomer	Tenuifolin [67]	Alzheimer's disease	A*β*-induced apoptosis in SH-SYHY cells
	Schisandrin A [68]	Parkinson's disease	1-methyl-4-phenyl-1,2,3,6-tetrahydropyridine-induced male C57BL/6 model
	Celastrol [69]	Parkinson's disease	1-methyl-4-phenyl-1,2,3,6-tetrahydropyridine-induced SH-SY5Y cells and mouse model
	Salidroside [70]	Parkinson's disease	1-methyl-4-phenyl-1,2,3,6-tetrahydropyridine-induced model in male C57BL/6 mice and MN9D cells
	Carnosic acid [71]	Parkinson's disease	6-hydroxydopamine-induced SH-SY5Y cells

**Table 2:** Liver protection of natural products through the regulation of PINK1.

Type	Natural products	Disease	Experimental models
Mixture	Zhiganfang [72]	Nonalcoholic steatohepatitis	High-fat diet-induced SD rats
Monomer	Cyanidin-3-O-glucoside [31]	Nonalcoholic fatty liver disease	High-fat diet-induced mice, palmitic acid-induced AML-12 cells and HepG2 human hepatocarcinoma cells
	Genipin [73]	Hepatic ischemia and reperfusion injury	Ischemia and reperfusion-induced hepatic injury in C57BL/6 mice
	Quercetin [74]	Non-alcoholic fatty liver disease	High-fat diet-induced C57BL/6 model and andoleate/palmitate-induced HepG2 cells
	Matrine [75]	Liver cancer	HepG2 and Huh7 cell lines
	Ginsenoside Rg1 [76]	Nonalcoholic fatty liver disease	Oleic acid-induced HL-7702 cells

**Table 3:** Anticancer activity of natural products through regulation of PINK1.

Natural products	Disease	Experimental models
Alantolactone [77]	Liver cancer	HepG2 cells
Tanshinone I [77]	Liver cancer	HepG2 cells
Polyphyllin I [78]	Breast cancer	Human breast cancer cells (MDA-MB-231 and MCF-7) and human mammary stromal cells (Hs-578Bst)
Ginsenoside Rh2 [79]	Breast cancer	Human breast epithelial cell line MCF-10A and breast cancer cell line MCF-7-GFP stable cells
Ursolic and Oleanolic Acids [80]	Lung cancer	A549 human lung cancer cells
Chalcomoracin [81]	Breast cancer	Human breast cell MDA-MB-231 and female nonthymic nude mice
Chalcomoracin [81]	Prostate cancer	Prostate cancer cells PC-3 and LnCAP

**Table 4:** Protection of myocardial injury by natural products through PINK1 regulation.

Type	Natural products	Disease	Experimental models
Mixture	Yimai Granule [82]	Hyperlipidemia combined with myocardial ischemia-reperfusion injury	High-fat diet combined with myocardial ischemia-reperfusion injury in SD rats
	Tongxinluo [83]	Myocardial ischemia-reperfusion injury	Left anterior descending coronary artery ligation performed for 50 min and reperfusion for 4 h for animal models of myocardial ischemia -reperfusion injury
	Shenmai Injection [84]	Myocardial ischemia and reperfusion injury	H9c2 cardiomyocytes subjected to 12 h hypoxia followed by 2 h of reoxygenation to induce cell injury
	Tongxinluo capsule [85]	Myocardial ischemia and reperfusion injury	Left anterior descending artery ligation and surgery, with 50 min ischemia followed by 4 h reperfusion in SD rats.
	Gualou Xiebai Banxia decoction [86]	Myocardial ischemia reperfusion injury	Reversible ligation of the left anterior descending coronary artery for 30 min and reperfusion for 2 h to prepare animal models of myocardial ischemia-reperfusion injury
Monomer	Gerontoxanthone I [87]	Myocardial ischemia and reperfusion injury	H9c2 cells incubated in a hypoxic chamber with ischemia-mimetic solution, then transferred into a normoxic incubator with fresh DMEM to establish the model
	Macluraxanthone [87]	Myocardial ischemia and reperfusion injury	H9c2 cells incubated in a hypoxic incubator with ischemia-mimetic solution, then transferred into a normoxic incubator with fresh DMEM to establish the model

**Table 5:** Other diseases alleviated by natural products through PINK1 regulation.

Type	Natural products	Disease	Experimental models
Mixture	*Pueraria lobata* extracts [88]	Diabetic nephropathy	Cadmium combined with high-fat and high-sugar feed in mice
	*Astragalus mongholicus* Bunge and *Panax notoginseng* (Burkill) F.H. Chen [89]	Diabetic nephropathy	*In vivo* autophagy deficiency model established in C57BL/6 mice by streptozocin combined with a high-fat and high-sugar diet. *In vitro* autophagy deficiency model induced by high glucose in renal mesangial cells.
	Huangqi-Danshen decoction [90]	Diabetic nephropathy	Male diabetic *db/db* mice and nondiabetic littermate *db/m* mice
Monomer	Astragaloside IV [91]	Diabetic nephropathy	Male diabetic *db/db* mice and nondiabetic littermate control *db/m* mice
	Mangiferin [92]	Obesity	C3H10T1/2 mesenchymal stem cells and human adipose-derived MSCs
	Curcumin [93]	Acute kidney injury	Cisplatin-induced injury model in rats
	Palmatine [94]	Ulcerative colitis	Dextran sodium sulfate-induced ulcerative colitis in mice. NLRP3 inflammasome activation in THP-l cells

## Abbreviations

hPINK: Human PINK1
MTS: Mitochondrial targeting sequence
OMS: Outer membrane localization sequence
TMD: Transmembrane domain
KD: Kinase domain
CTE: C-terminal extension
NMR: Nuclear magnetic resonance
TcPINK1: Tribolium castaneum PINK1
PhPINK1: Pediculus humanus corporis PINK1
UB: Ubiquitination
LC3: Light chain 3
PD: Parkinson's disease
ROS: Reactive oxygen species
NSCLC: Non-small-cell lung cancer
CDDP: Cisplatin
HCC: Hepatocellular carcinoma
MAFLD: Metabolic-associated fatty liver disease
DAMPs: Damage-associated molecular patterns
IR: Ischemia/reperfusion
AMPK: AMP-activated protein kinase
*ΔΨ*m: Mitochondria transmembrane potential
TRAP-1: TNF receptor-associated protein 1
THPostC: Thyroid hormone postconditioning
TSG101: Tumor susceptibility gene 101
PTEN: Phosphatase and tensin homolog
APE1: Apurinic/apyrimidinic endonuclease 1
RCOL: *Rhodiola crenulata* oral liquid.
